# Analysis of Functional Recovery and Subjective Well-Being after Arthroscopic Rotator Cuff Repair

**DOI:** 10.3390/medicina57070715

**Published:** 2021-07-15

**Authors:** Aušra Adomavičienė, Kristina Daunoravičienė, Rusnė Šidlauskaitė, Julius Griškevičius, Raimondas Kubilius, Lina Varžaitytė, Juozas Raistenskis

**Affiliations:** 1 Department of Rehabilitation, Physical and Sports Medicine, Faculty of Medicine, Vilnius University, Santariskiu g.2, LT-08661 Vilnius, Lithuania; rusne.sidlauskaite@gmail.com (R.Š.); juozas.raistenskis@santa.lt (J.R.); 2 Department of Biomechanical Engineering, Vilnius Gediminas Technical University, J. Basanaviciaus Str. 28, 03224 Vilnius, Lithuania; kristina.daunoraviciene@vilniustech.lt (K.D.); julius.griskevicius@vilniustech.lt (J.G.); 3 Rehabilitation Department, Lithuanian University of Health Sciences, Eiveniu Str. 2, LT-50161 Kaunas, Lithuania; Raimondas.Kubilius@kaunoklinikos.lt (R.K.); lina.varzaityte@gmail.com (L.V.)

**Keywords:** rotator cuff repair, hand motor function, rehabilitation, subjective well-being, long-term context

## Abstract

*Background:* Rotator cuff tears are common causes of functional shoulder instability and often lead to arthroscopic rotator cuff repair. A well-programmed rehabilitation leads to successful tendon healing, positive functional recovery and subjective well-being (SWB). *Objective:* To evaluate the changes in shoulder functioning and SWB pre-, post-outpatient rehabilitation and after one-month follow-up. *Materials and Methods*: A total of 44 patients were assessed three times: at the beginning (six weeks’ post-surgery), at the end of outpatient rehabilitation (2–3 weeks) and one month after rehabilitation. The outcome measures were the Disabilities of the Arm, Shoulder and Hand score (DASH), active range of motion (ROM), manual muscle testing (MMT), hand dynamometry (HD) and pain level by a Visual Analogue Scale (VAS). SWB was assessed by Rosenberg self-esteem scale (RSES), Positive and Negative Affect Schedule (PANAS) and the Lithuanian Psychological Well-Being Scale (LPWBS). Results are presented as a difference between periods. *Results:* Affected shoulder motor function (MMT, HD and ROM) significantly improved in three periods (*p* < 0.05); however, major recovery was observed in the follow-up period. VAS scores meaningfully decreased over all stages and negatively correlated with motor function recovery (*p* < 0.05). DASH rates exhibited significant retrieval in all phases, especially in follow-up. SWB results demonstrated the larger effects of self-evaluation in follow-up, improved daily functions and psychological wellness, then negative emotions significantly decreased (*p* < 0.05). *Conclusions:* The experienced pain and psychosocial factors significantly influence functional recovery of the shoulder during rehabilitation. The improvement in motor function, ability and pain relief during rehabilitation increases level of SWB, psychological wellness and positive emotional affect in long-term context.

## 1. Introduction

More than 1.7 billion people, nearly 25% of the world’s population, are affected by a musculoskeletal condition [1]. Rotator cuff (RC) tear trauma, the most common shoulder pathology, accounts for 35–50% of all diagnoses of shoulder injuries, especially among middle-aged and elderly people, and ≥40% of individuals 60 years or older have this condition [2,3]. In as many as 74% of cases, this type of injury is identified as a major cause of shoulder pain, causing changes in the structure of muscles and tendons and disrupting the biomechanics of shoulder [4,5].

RC pathology can result in weakness, shoulder instability and limitation of daily activities (work and sports). Symptomatic disease affects between 4–32% of the patient population with RC tears. Although patient’s age, activity level, size of the tear and smoking status influence the decision of management, and the frequently preferred method of initial treatment is non-surgical. However, if this management is unsuccessful, surgical repair has been shown to relieve pain and improve function in >90% of patients [6]. People with RC tears very often experience significant changes in their ability to be independent in their daily and professional activities and are often forced to undergo shoulder surgery due to the resulting pain, decreased muscle strength and range of motion (ROM) [7]. Arthroscopic debridement or partial RC reconstruction achieves good results in shoulder joint function [5], but massive shoulder dysfunction does not always produce good treatment and functional recovery results and unfortunately, significant limitations of shoulder function remain present [5,6]. Patients often have a long and painful post-operative period, characterized by decreased psychological well-being, ROM, muscle atrophy, impaired proprioception and daily activities and negative thinking and less physical activity are associated with greater shoulder pain and disability [8]. Higher levels of psychological stress after RC tear surgery are increasingly associated with poorer outcomes for initial patient shoulder pain and functional status [9]. According to previous research, a successful outcome is very dependent on surgical technique, as it is on rehabilitation [10]. There are a number of different rehabilitation protocols for RC tear, and they all aim to do the same—protect the shoulder—while increasing ROM and muscular endurance and strength to return to daily activities [7], and decreasing pain and improving the quality of life [11]. Mostly, rehabilitation protocols for an RC tear does not differ much regarding methods, but may differ in the period of performance—early passive motion, starting as soon as possible postoperatively, and delayed active motion, usually starting at five to six weeks postoperatively [12]. The traditional rehabilitation protocol, consisting of a two-week period of procedures after four to six weeks of immobilization, has been proven to result in excellent outcomes immediately after surgery and at one-year follow-up [13]. It has also been documented that a close communication between the surgeon, patient and physical therapy team is important and should continue throughout the entire recovery process [10]. However, it has been observed that patient satisfaction with their condition and how it affects their lives after RC surgery starts to significantly improve after 2.5 months, ranging to six months. Meanwhile, clinically relevant functional status results for patients are usually achieved only after a median postoperative period of two years [14,15,16]. Every patient who undergoes shoulder surgery for the treatment of RC tear is commonly assessed pre- and postoperatively with general and shoulder-specific measures of pain and function. Unfortunately, there is a paucity of scientific papers analysing the relationships between psychological well-being and patient-reported outcomes after arthroscopic RC repair, to the degree in which the decrease or increase of a patient’s psychological well-being could be associated with differences in functional outcomes after recovery from this condition in the follow-up period, especially a short-term one [17].

Potter et al. state that patients with shoulder and RC pathology who exhibit greater levels of psychological distress report inferior preoperative self-assessments of pain and function [6]. Mild to moderate levels of distress did not diminish patient-reported outcomes to an important clinically degree in this small series of patients with RC tears [18]. Higher levels of psychological distress are also correlated with decreased patient-reported outcomes after surgical intervention. Greater levels of pre- or postoperative psychological distress and subjective well-being (SWB) were not associated with clinically important differences in outcome scores (visual analogue scale (VAS) for pain, hand functionality-evaluated scores) one year after arthroscopic RC repair; similarly, higher levels of distress were not associated with less interval improvement in outcome scores at one year [19,20]. RC tears are a quite common condition that is often incapacitating.

Allowing healing of the repaired RC tendon while minimising stiffness and muscle atrophy are the primary goals of postsurgical rehabilitation. Both protective and accelerated rehabilitation protocols must be utilised based on individual patient risk for developing postoperative hand muscle stiffness (muscle strengthening exercises are delayed and applied after arthroscopic repair of RC tears greater than 5 cm or involving more than two tendons, poor tissue quality or repairs with greater tension). Arm position during immobilisation (four to five weeks after surgery) is also of importance during the early healing phase of tendon repair [14,16]. Continuous passive motion, cryotherapy or joint mobilization post-surgically is used to minimise postoperative pain, swelling, muscle spasm, inflammatory response while maintaining ROM following articular cartilage treatments and joint arthroplasty. Rehabilitation protocols are frequently divided into four phases, progressing from a maximum protection phase to a minimum protection phase [13,17]. The patient is advanced to the next phase once the patient has achieved passive and active ROM goals, in addition to meeting the established timelines required for progression, and the patient is consistently trained/educated [5,18]. Although the outcome of surgical procedures for RC repair is, in fact, generally satisfactory, patients have to be carefully assisted in their postoperative period, must be provided adequate and straightforward patient education and gradually applied rehabilitation measures in order to guarantee a good final rehabilitation program and long-term context outcomes [18].

We raise the hypothesis that an outpatient rehabilitation program can be more effective in reducing pain and improving primary recovery of shoulder motor function (early passive ROM, muscle strength, etc.), but arm functionality in daily life and SWB significantly improves more long-term and depends on patient responsibility and achieved rehabilitation outcomes.

The objectives of the study are to clarify the period during which the highest recovery in different areas of shoulder motor function has been observed and how much this impairment affects SWB.

## 2. Materials and Methods

### 2.1. Selection and Description of Participants

According to strict inclusion criteria, a small number of patients that underwent rehabilitation in our outpatient unit in a prospective cohort clinical trial were involved (a total of 60 patients undergoing outpatient rehabilitation program after arthroscopy of RC tears). Patients underwent arthroscopic RC repair and after 5–6 weeks began participation in a 2 weeks’ duration conventional outpatient rehabilitation (physical therapy, occupational therapy, massage, physiotherapy, psychologist consultation and patient’s education). Long-term data collection was completed at the scheduled follow-up one-month after rehabilitation.

Inclusion criteria identified patients who (1) were ≥ 40 years old and scheduled for a shoulder arthroscopy for a primary symptom of shoulder pain secondary to a reparable full-thickness RC tear, (2) underwent surgical intervention for the first time, (3) started an outpatient rehabilitation program six to seven weeks after surgery and (4) attended follow-up one month (30–35 days) after rehabilitation. Exclusion criterion included: (1) young patients (<39 years old), (2) RC tear retraction and repeated shoulder arthroscopy, and (3) outpatient rehabilitation started more than seven weeks after surgery.

Five patients (8%) did not complete the outpatient rehabilitation program and 11 patients (25%) did not return for scheduled follow-up after one month due to it being their second assessment completed, due to worsening health (increased pain, high blood pressure or heart rate problems or intolerance of physical load). In total, 44 of 65 patients (67%) completed the study and were included in the analysis of final data. Patients who did not return for follow-up after one month were also contacted by phone, mail or email and asked to complete questionnaires to improve the follow-up rate. The assessment data was collected, scored and entered into a database by a study coordinator.

### 2.2. Characterisation of Population

The average age ± standard deviation (SD) of 44 patients who participated in all three stages of the study was 57.4 ± 2.8 years, with 28 men (63.64%) and 16 women (36.36%). The main sociodemographic characteristics of participants are presented in Table 1. The main reasons for RC tears were unknown and related to degenerative RC muscle changes. Of the patients, 23 (52.27%) stated that movements of the shoulder were painful for a long time, 16 (36.36%) patients related pain due to nature of working position and in five (11.36%) patients, injury was caused by trauma while participating in sports (Table 1). 

### 2.3. Description of Follow-Up Routine

The main outcome measures used for evaluation of hand motor function recovery after RC tears were the following: Disabilities of the Arm, Shoulder and Hand score (DASH), which is a comprehensive self-administered questionnaire about the symptoms and functioning of the entire upper extremity [20]; manual muscle testing (MMT), used as a method of measuring upper extremity muscle strength [21,22]; hand grip muscle strength, tested by hydraulic hand dynamometry (HD, in kg) to obtain kinematic parameters of requested movements; and active ROM of the shoulder and elbow were measured [13]. As a psychometric response scale and measurement instrument for the subjective states of intensity, severity, and nature of experienced pain was used VAS scale [2]. For evaluation of the changes of patients’ SWB, the following instruments were used: patient’s self-esteem was evaluated by the Rosenberg self-esteem scale (RSES) [23], positive and negative affect was evaluated using the Positive and Negative Affect Schedule (PANAS) [24] and psychological well-being was assessed with the Lithuanian Psychological Well-Being Scale (LPWBS, the authors’ consent to use the scale in the study was obtained) [25]. Assessments were extracted at the beginning (pre-outcomes/I period) and the end (post-outcomes/II period) of outpatient rehabilitation and as follow-up outcomes after one month (III period).

### 2.4. Statistics

The Lilliefors normality test (*p* < 0.05) was used to test for data normality. The results for the three periods were compared in the following order: the I with the II period, and then the II with the III period. Normally distributed data were compared utilizing the parametric statistical method, i.e., paired two-sample *t*-test (*p* < 0.05). Not normally distributed data (*p* < 0.05) were compared employing a non-parametric statistical method, i.e., the Wilcoxon signed rank test (*p* < 0.05). The normally distributed data are represented as mean ± SD, while the non-normally distributed data are represented by median (MAD, IQR). The Cohen’s value d was calculated to evaluate effect size: d = 0.01—very small effect size, d = 0.20—small effect size, d = 0.50—medium effect, size, d = 0.80—large effect size, d = 1.20—very large effect size, d = 2.00—huge effect size [26]. With regard to the relationship between quantitative variables, Spearman’s correlation coefficient (r) was computed. A correlation coefficient of r < 0.3 was considered low while 0.3 ≤ r ≤ 0.7 was considered moderate and r > 0.7 was considered high. We set the significance level at *p* < 0.05. All the analyses were carried out using MatlabR2019b software (MathWorks Inc, Portola Valley, CA, USA).

## 3. Results

**Figure 1:**
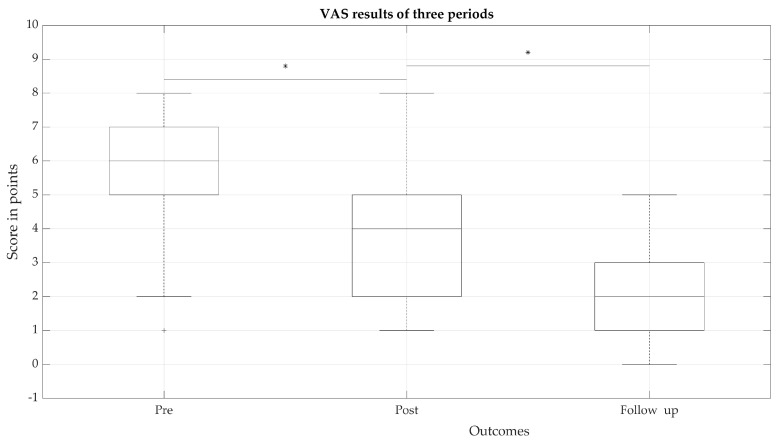
VAS scale (*n* = 44) variation. * significant differences in periods with *p* < 0.05.

VAS psychometric response scores for the subjective states of intensity, severity and nature of experienced pain (Figure 1) were significantly different for the three periods (*p* < 0.05). A significant decrease over all periods indicating that pain decreased with the use of rehabilitation measures and their long-term effects were observed in the follow-up outcomes, resulting in the eventual decrease of pain. However, it can be observed that in the follow-up period, the pain experienced by patients was on average four points lower, compared to pre-period.

The estimates of pain experienced by most patients in the post-stage decreased significantly compared to pre-stage, but several (11%) patients remained with pain estimates that continued to be high (reported > five points), with even the highest reported patient pain in the follow-up period not exceeding five points.

**Figure 2:**
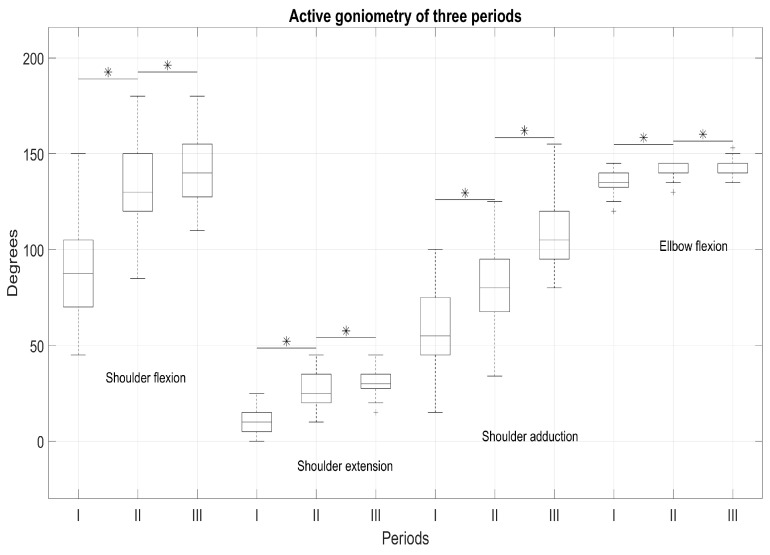
Ranges of Motion (ROMs) of shoulder and elbow in I-II-III periods. * significant differences in periods with *p* < 0.05.

The distribution of subjects according to affected hand side showed that the affected right hand prevailed in 35 (79.5%) subjects and the affected left hand in nine (20.45%) subjects. Affected hand grip strength significantly (*p* < 0.05) differed in the three periods with effect size d = 1.93 for pre- and post-stage; d = 2.1 for post-stage/follow-up outcomes and, for follow-up/healthy hand, d = 1.42 (Table 2 and Figure 2). However, the largest recovery was observed in the follow-up period, although strength did not recover and did not reach the average of a healthy hand (Table 2). We also observed that dominant (right) and non-dominant (left) hand grip strength recovery did not differ significantly in all periods; total recovery of the affected left hand was 12.78 kg and 12.23 kg for the affected right hand.

Meaningful results of the effect size kinematic parameters of the requested movements of the shoulder and elbow (measured by MMT) and joint mobility (active goniometry) in the three periods are presented in Table 3 and Table 4. It was observed that the kinematic parameters of the shoulder and elbow differed distinctly from I to II periods, and from II to III periods, a weak difference effect was observed, especially for the elbow segment.

Our study showed that a patient’s shoulder and elbow muscle, hand grip strength and hand ROM significantly improved over all three periods; however, a significantly decrease in pain was also observed. The significant reduction in pain negatively correlated with hand grip strength (HD) (r = −0.38, *p* = 0.01) and elbow flexion (r = −0.298, *p* = 0.049). Furthermore, increasing ROM showed better correlations to pain in II and III periods, which indicated effective complex rehabilitation measures that reduced pain and increased arm motor function.

**Figure 3:**
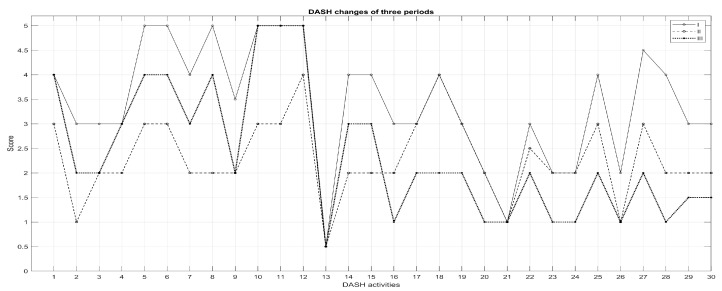
DASH scores of I, II and III periods (*n* = 44).

According to results of the Disabilities of the Arm, Shoulder and Hand (DASH) questionnaire, a comprehensive self-report questionnaire in which patients can rate difficulty and interference in daily life to perform certain upper extremity activities on a five-point Likert scale, meaningful changes in the answers in three periods were revealed. However, the study results show different recovery of an individual ability to complete tasks, for which the patient can select an appropriate number corresponding to his/her severity/function level. Figure 3 demonstrates the effect size of each question (*n* = 30) response. Outcome effect from I to II period demonstrated very high and significant effect size in the first half of the questionnaire. However, the period from II to III showed smaller various effect sizes, in comparison to the previous term. The scores on both tests ranged from 0 (no disability) to 100 (most severe disability). Lower scores indicated a lower level of disability. At I period, the average DASH total score was 105.27 ± 9.31 points, at II period, 68.45 ± 8.50 points and finally, at III period, the average score was 72.68 ± 6.73 points. A statistical difference was found between all DASH questions in the periods, except for 21 between periods II and III and 22 between periods I and II (Figure 3).

Patients self-reported the disabilities of the upper extremity following the DASH 1–21 activities (presented in Table 5) and results showed that most patients had severe difficulty or were unable to perform activities such as lifting an object, manipulating hands, doing housework and shopping. Results also showed many difficulties in service activities at the beginning of rehabilitation, but participation in these activities improved at the end of rehabilitation, although moderate difficulties remained in most activities. Unfortunately, during the follow-up period, the predominance of mild difficulty was observed in most activities, mainly in service and leisure activities. Social functions and the ability to participate in community or vocational fields mostly recovered in the follow-up outcomes period (Table 5).

Patients self-reported pain, tingling, weakness, stiffness and other discomfort related to affected upper extremity following activities 24–30, with these symptoms demonstrated as moderate in I period, then decreased to mild or non-symptoms in II or III periods (Table 6).

**Figure 4:**
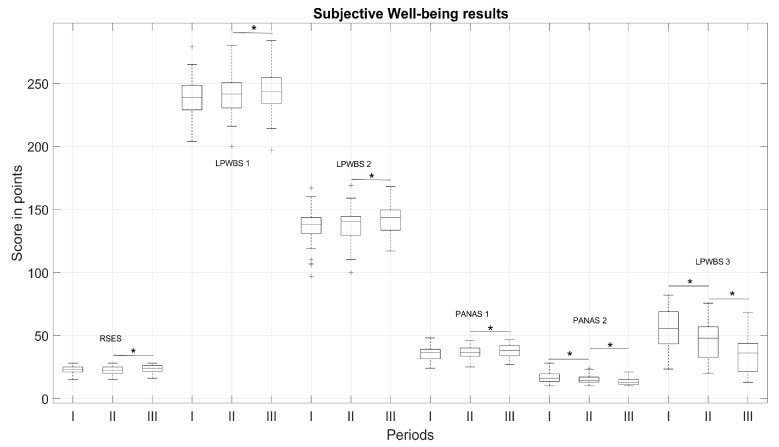
Subjective Well-being evaluation results (*n* = 32): Self-esteem by Rosenberg self-esteem scale (RSES); Psychological wellness (Lithuanian Psychological Well-Being Scale, LPWBS 1), Life satisfaction score (LPWBS 2), Positive (Positive and Negative Affect Schedule, PANAS 1) and Negative Affect (PANAS 2), Daily functions (LPWBS 3). * Significant differences in periods with *p* < 0.05.

In the study, the changes of SWB following RC tears were analysed and are displayed in Figure 4. A total of 32 patients completed the fully electronic questionnaire in III period. Several of them were unable to perform that independently and were excluded from final analysis.

A correlation analyses demonstrated that the level of experienced pain was directly related with patients’ positive and negative affect. In I and II period, higher VAS level determined lower positive affect and higher negative affect; however, improvement in motor function, participation in activities of daily living (ADLs) and reduction in pain, thus increasing the level of positive affect and decreasing negative affect (Table 7).

Overall results demonstrated that the subjective variables of symptoms and function have the most robust associations with SWB following RC tears. The larger effects of self-evaluation were indicated for the post- and follow-up periods. The results of patients’ self-esteem by measured Rosenberg self-esteem scale (RSES) and Lithuanian Psychological Well-Being Scale (LBWS) scores revealed significantly different results only in the follow-up outcomes (*p* < 0.05). Self-esteem by the RSES from I to II period increased by the small effect size (from 22.53 ± 3.42 to 22.13 ± 3.14, d = 0.29 (small effect)) and the meaningful changes were observed from II to III period (from 22.13 ± 3.14 to 23.53 ± 3.13 *, d = −1.03 (large effect) *), which indicate an increasing higher global self-esteem level. Psychological wellness increased significantly from I to II period (from 239.09 ± 15.10 to 240.31 ± 15.37, d = −0.34 (small effect), but a large effect was significantly lower than for II/III period (from 240.31 ± 15.37 to 243.44 ± 17.51 *, d = −0.53 (medium effect) *). Life satisfaction score for I/II period displayed small changes (from 135.38 ± 15.06 to 137.03 ± 14.75), in comparison to II/III (from 137.03 ± 14.75 to 141.16 ± 13.35 *, d = −0.58 (medium effect) *). The PANAS showed similar tendency of outcomes variation: the size of negative emotions significantly decreased more in post-/follow-up (for pre-/post-, values decreased from 16.59 ± 4.76 to 15.22 ± 3.86 * (0.98; large effect)) * and for post-/follow-up, values dropped to 13.06 ± 2.77 * (d = 1.20 (large effect) *, *p* < 0.05). Subsequently, positive emotions significantly increased in post-/follow-up (for pre-/post-, values increased from 36.00 ± 5.53 to 36.41 ± 5.53 (d = −0.27 (small effect)) and for post-/follow-up period, values increased to 37.78 ± 5.05 * (d = −0.72 (medium effect) *, *p* < 0.05). Daily function scores varied the same way as negative emotions (decreased from 54.12 ± 17.30 to 46.80 ± 16.24 * (d = 1.71; very large effect*) for pre-/post- and to 35.36 ± 14.90 * (d = 2.33; huge effect) * for post-/follow-up).

## 4. Discussion

To ensure that the rehabilitation measures are effective, the outcomes after arthroscopic RC repair should be evaluated over time. What determines the effectiveness of rehabilitation? It is quite clear that both the complexity of the surgical intervention [5,6,8,18] and the means of rehabilitation and motivation of the patient [7,9,10,11] are most significant. In our study, the improvements of arm/hand motor function and SWB of the patient were collected over time in all stages, even though the long-term period in our study was relatively short compared to other studies over a 6-, 12- or 48- month follow-up period [10,26]. Although the results were sustained over time, after 6 or 12 months, residual events and dysfunction persisted [14,15,16]. Therefore, it is very clear that patient recovery rates need to be monitored and evaluated for a longer period of time. [10]. The most important indicator, which is named by the patient both before and after the surgical period, is pain [24,25]. The pain we evaluate in our study is very related to the condition of the tissues, that is degeneration of soft tissues/tendons and leads to the surgery [27,28]. In the frame of our study, significant improvements were noted in pain relief in all periods (*p* < 0.05). Pain became the key factor for our further study, as we correlated it with the almost all obtained parameters and indicated the most sensitive. The use of rehabilitation measures and their long-term effects were mostly observed only in the follow-up outcomes. Furthermore, in follow-up period, the pain experienced by patients was lower compared to the pre-period, and it also affected the recovery of movements, strength and hand functionality. The higher level of experienced pain in pre- and post-rehabilitation period could be influenced by the fact, that more our patients had a massive damage of m.supraspinatus (45.45%) and combined muscle (m.supraspinatus and m.infraspinatus) (26.54%). Affected hand grip strength significantly (*p* < 0.05) differed in three periods; however, we observed that dominant (right) and non-dominant (left) hand grip strength recovery did not differ meaningfully. The largest recovery in the global shoulder evaluations (kinematic parameters, ROM and muscle strength) was also observed in the follow-up period, and compared to the strength of a healthy hand, strength did not recover and did not reach the average of a healthy hand. Furthermore, an increase of ROM shows a better correlation to pain relief in II and III periods, which confirms that integrated rehabilitation measures are effective in reducing pain and restoring arm motor function. The overall success and effective recovery of motor function depends on the rehabilitation treatment protocol, which is characterised by the requirement of some supervised physical therapy, often requiring 12 weeks or more, focusing on supine exercises with gradual progression to upright [7]. To obtain significant improvements in strength and ROM, the physical exercises used in the rehabilitation program should be continued as a home exercise program [6]. Consequently, it is very important to provide the rehabilitation measures for pain relief during rehabilitation and the long-term period [6,10].

According to the self-report questionnaire DASH, in which patients can rate difficulty and interference in daily life to perform certain upper extremity activities, this study’s results showed different recoveries of individual abilities to complete tasks (Table 5 and Table 6). At the beginning of rehabilitation, most patients had severe difficulty or were unable performing activities such as lifting an object, manipulating hands, doing housework and shopping. Nevertheless, participation in these tasks improved at the end of rehabilitation, although moderate difficulties remained in most activities. A lower level of disability was detected during the follow-up period, but, unfortunately, the predominance of mild difficulty was still observed, mainly in daily and leisure activities. Negative thinking and less physical activity in daily life were associated with permanent shoulder pain during activity caused by muscle atrophy, restricted movements and shoulder stiffness [8].

Patient SWB following RC tears has been associated with pain, dysfunction, functional difficulty and outcome after RC surgery [19,24]. In our study, the changes of subjective satisfaction and overall rehabilitation results established the most robust associations. A correlation analysis demonstrated that the level of experienced pain directly related to patients’ positive and negative effects, and during rehabilitation, the higher VAS level determines higher negative effect (Table 7). However, improvement in motor function increased participation in daily life activities. Moreover, the reduction of pain may consequently decrease a negative effect and lift the level of positive emotions. Complex rehabilitation measures and their long-term effects were observed more in the follow-up outcomes, but subjective satisfaction and psychological wellness of patients was higher at the end of rehabilitation, possible since patients subjectively felt functional improvement and were more motivated. In the follow-up period, patients’ subjective wellness stabilised and outcomes were not changed. This indicates that when assessing a patient’s progress at all stages, it is important to consider the patient’s subjective assessment, as well as compare the patient’s assessment with the recovery of motor function. We also noted other signs of patient mental state and motivation, as well as manifestations of influence in the restoration of motor functions. However, we cannot yet confidently confirm these facts, because the limitations of our study, such as a large (33%) loss of participants and finally the small sample size of the study, the abundance of parameters, and the relatively short period of time, do not allow us to do so. We also assume that the results of muscle strength studies could change by reorganizing the study cohort. For now, the goal was to evaluate the decrease/increase in strength in the affected arm during rehabilitation periods. We also took into account and compared the result of dominant and non-dominant hand. However, we believe that the breakdown of patients by gender would allow us to look at the outcome even differently and find new changes and trends. We also suggest, if possible, the inclusion of pre-operative examination into the protocol, because it would strongly supplement prognostic indicators of motor shoulder joint recovery. Meanwhile, we are pleased to have been able to confirm our hypothesis at this stage of the study and select the most important parameters for assessing the progress of motor function recovery after arthroscopic RC repair and evaluation of patients’ psychoemotional state and SWB affected this dysfunction. It is very important to state, that our used methods, regarding cost-effectiveness, are easy to use, simple and practicing the self-reported identification of main problems. They can help the rehabilitation team and patients to choose the right techniques, the right physical activity and reduce disability. These findings suggest the need to explore and carefully plan strategies to stimulate positive improvement within rehabilitation.

## 5. Conclusions

This study found that the experienced pain and psychosocial factors after RC tears do significantly influence the level of disability and functional recovery of shoulder. Patient expectations and outcomes significantly increased during the rehabilitation program, with decreasing experienced pain, and patients had higher positive emotional effect and significant shoulder motor function recovery. However, shoulder functioning and the ability to perform daily living activities in the long-term period reached the highest recovery, although the positive emotional effect did not change significantly. The follow-ups after one month showed that a home exercise program must be continued consistently, as an intensive three- to four-week rehabilitation program is not sufficient to significantly improve shoulder function and functioning in ADLs and reduce the psychosocial confounders of patients. Most importantly, improvement in motor function and the ability to perform ADLs are sensitive to pain level. Pain relief directly positively affects a high level of SWB, psychological wellness, and positive emotional affect after RC tears.

## Figures and Tables

**Table 1:** Sociodemographic characteristics of participants (*n* = 44).

Sociodemographic Characteristics of Participants		
Age, mean (Standard Deviation, SD)	57.4 ± 2.8 years (min-max 40–65 years)	
Gender	28 (63.64%) men/16 (36.36%) women	
Education	Primary 9 (20.4%)/Secondary 13 (29.5%)/Higher education 22 (50%)	
Work status	Not working 15 (34%)/Working 26 (59.1%)/Partly working 3 (6.8%)	
Duration of shoulder complaint	<3–6 months 23 (52.3%)/6–12 months 11 (25%)/12–24 months 6 (13.6%) > 24 months 4 (9.09%)	
Dominant arm affected	Yes 36 (81.8%)/No 8 (18.2%)	
	Affected Left hand (*n* = 9)/Right hand (*n* = 35)	
Damaged muscle	*n* (%)	
m. supraspinatus	20 (45.45%)	
m. infraspinatus	6 (13.63%)	
combined (m. supraspinatus and m. infraspinatus)	13 (26.54%)	
m. subscapularis	5 (11.37%)	
m. teres minor	0 (0%)	
Circumstance of the injury	Number	Percent, %
Fall from a height	0	0
Heavy weightlifting	9	20.45
Collapse (from cycling, walking, running, rushing)	4	9.09
Making a sudden movement over the shoulder	5	11.36
Trauma during sports	5	11.36
Constant repetitive load on the shoulder	13	29.55
Do not know, it just hurt more and more	8	18.18

**Table 2:** The parameters of hand grip strengths (kg, means ± standard deviation).

Affected Left Hand (*n* = 9)	Pre-Outcomes (I)	Post-Outcomes (II)	Follow-Up Outcomes (III)	Healthy Arm (Left Hand)
Means ± SD	25.00 ± 5.92	30.56 ± 6.69	37.78 ± 8.60	49.89 ± 13.97
Difference in periods	5.56			NA
		7.72		
Affected Right hand (*n* = 35)	Pre-outcomes (I)	Post-outcomes (II)	Follow-up outcomes (III)	Healthy arm (Right hand)
Means ± SD	24.60 ± 8.71	30.80 ± 9.70	36.83 ± 10.86	47.80 ± 14.78
Difference in periods	6.20			NA
		6.03		

Values presented as means ± standard deviation; Differences reflect the effect obtained by subtracting the results for the periods: “post-” minus “pre-” and “follow-up” minus “post-” and total effect “follow-up” minus “pre-”. NA—not applicable.

**Table 3:** Manual Muscle Testing (MMT) results (*n* = 44).

Parameter	Pre-Outcomes (I)	Post-Outcomes (II)	Follow-Up Outcomes (III)
Shoulder flexion, score	3 (2.54–3.46)	4 (3.42–4.13)	5 (4.18–4.86)
Effect size, *d*	1.42		
		1.48	
Shoulder extension, score	3 (2.76–3.51)	4 (3.71–4.25)	4 (3.93–4.48)
Effect size, *d*	1.96		
		0.48	
Shoulder adduction, score	3 (2.54–3.36)	4 (3.52–4.12)	4 (3.82–4.41)
Effect size, *d*	1.87		
		0.58	
Shoulder abduction, score	3 (2.62–3.60)	4 (3.55–4.13)	4 (3.93–4.48)
Effect size, *d*	1.46		
		0.75	
Elbow flexion, score	4 (3.42–4.03)	4 (3.92–4.45)	4 (3.95–4.51)
Effect size, *d*	0.83		
		0.22	
Elbow extension, score	4 (3.45–4.05)	4 (3.91–4.41)	4 (3.98–4.61)
Effect size, *d*	0.75		
		0.39	

* Significant differences with *p* < 0.05, in periods highlighted/bolded. Values presented as medians with Interquartile range (IQR) range.

**Table 4:** Active goniometry results (*n* = 44).

Parameter	Pre-Outcomes (I)	Post-Outcomes (II)	Follow-Up Outcomes (III)
Shoulder flexion, °	90.80 ± 29.21	130.57 ± 25.02	140.00 (129.61–154.26)
Effect size, d	2.49		
		0.89	
Shoulder extension, °	10 (6.97–14.84)	25 (20.45–32.50)	30 (26.78–37.09)
Effect size, d	2.54		
		0.90	
Shoulder adduction, °	55 (44.11–72.02)	80 (66.84–94.94)	105 (95.97–118.81)
Effect size, d	2.59		
		2.20	
Elbow flexion, °	135 (130.06–139.48)	145 (138.43–144.53)	145 (141.51–146.12)
Effect size, d	1.13		
		0.48	

* Significant differences with *p* < 0.05, in periods highlighted/bolded. Values presented as means ± SD or as medians with IQR range.

**Table 5:** Distribution of patients answers according to Disabilities of the Arm, Shoulder and Hand (DASH, 1–23 activities).

DASH Activities	Pre-Outcomes/Post-Outcomes/Follow-Up Outcomes (*n*, %)				
	1	2	3	4	5
	5-Point Scale				
1.	-/9.09/-	-/31.8/15.9	-/25/25	54.6/25/36.4	45.5/9.09/2.7
2.	63.6/40.91/13.6	22.7/34.9/47.73	36.36/2.27/11.3	22.73/-/-	4.55/-/-
3.	11.3/45.4/27.3	22.73/36.3/29.55	22.73/18.1/31.8	27.27/-/4.55	15.9/-/6.82
4.	-/27.2/29.55	22.73/38.6/36.3	29.55/31.8/13.6	25/22.7/6.82	22.7/13.64/-
5.	-/2.27/4.55	-/29.6/38.6	18.18/38.6/20.4	18.18/27.3/	63.6/50/36.3
6.	-/2.27/2.27	-/29.6/38.6	-/52.3/40.9	38.64/13.6/-	61.4/22.7/18.1
7.	-/25/25	-/38.6/38.6	20.45/31.8/31.8	36.36/4.55/-	43.2/-/4.55
8.	-/15.9/11.36	-/52.3/31.8	6.82/31.8/43.2	36.36/-/13.6	56.8/-/-
9.	-/34.1/54.6	-/18.2/29.6	50/18.2/6.8	40.91/2.3/-	9.09/9.09/-
10.	-/-/-	-/25/4.55	-/50/34.1	11.36/25/-	88.64/-/61.4
11.	-/2.27/-	-/2.95/4.55	-/43.8/29.6	11.36/25/-	88.64/-/65.9
12.	-/-/-	-/9.9/13.6	-/25/29.6	13.64/59.9/-	86.36/-/56.8
13.	50/50/50	11.36/31.8/11.36	2.27/15.9/6.82	22.7/2.27/13.6	13.64/-/18.2
14.	-/36.6/-	-/43.8/-	20.45/18.18/50	38.6/13.6/22.7	40.9/6.82/9.09
15.	-/38.6/-	-/43.2/-	25/29.55/43.2	43.2/18.2/22.7	31.82/-/4.55
16.	-/47.7/-	31.82/22.7/56.8	36.36/43.18/-	31.82/4.55/-	25/-/-
17.	-/25/-	2.27/47.7/56.8	18.18/43.18/-	34.09/22.7/4.5	45.45/-/-
18.	-/2.27/-	2.27/13.6/9.09	18.18/27.7/22.7	34.09/31.8/-	45.45/25/-
19.	11.4/20.5/-	29.55/27.3/45.45	27.27/25/27.3	20.45/18.2/-	11.4/9.09/-
20.	6.82/31.8/36.4	45.45/43.2/61.36	29.55/25/-	18.18/-/-	-/-/-
21.	63.6/79.5/2.27	22.73/15.9/86.36	13.64/4.55/2.27	-/-/-	-/-/-
22.	13.6/9.09/45.4	27.27/40.9/36.3	22.73/29.5/18.2	25/20.5/-	11.36/-/-
23.	29.5/43.2/68.1	38.6/36.4/25	13.6/15.9/4.55	15.9/4.55/2.27	2.27/-/-
DASH activities: 1. Open a tight or new jar. 2. Write. 3. Turn a key. 4. Prepare a meal. 5. Push open a heavy door. 6. Place object on a shelf above your head 7. Do heavy household chores. 8. Garden or do yard work. 9. Make a bed 10. Carry a shopping bag or briefcase. 11. Carry a heavy object (over 10 lbs). 12. Change a lightbulb overhead. 13. Wash or blow dry your hair. 14. Wash your back. 15. Put on a pullover sweater. 16. Use a knife to cut food. 17. Recreational activities which require little effort. 18. Recreational activities in which you take some force or impact through your arm, shoulder or hand. 19. Recreational activities in which you move your arm freely. 20. Manage transportation needs. 21. Sexual activities. 22. During the past week, to what extent has your arm, shoulder or hand problem interfered with your normal social activities with family, friends, neighbors or groups? 23. During the past week, were you limited in your work or other regular daily activities as a result of your arm, shoulder or hand problem?					

Abbreviations: Activities 1–21: 1—no difficulty, 2—mild difficulty, 3—moderate difficulty, 4—severe difficulty, 5—unable; Activities 21–23: 22 question: 1—not at all, 2—slightly, 3—moderately, 4—quite a bit, 5—extremely; 23 question: 1—not limited at all, 2—slightly limited, 3—moderately limited, 4—very limited, 5—unable.

**Table 6:** Distribution of patients answers according a rate the severity of the following symptoms ((DASH) 24–30 activities).

DASH Activities	Pre-Outcomes/Post-Outcomes / Follow-Up Outcomes (*n*, %)				
	1	2	3	4	5
	5-Point Scale				
24.	9.09/43.1/65.9	52.2/45.4/31.82	22.7/11.4/2.27	15.9/-/-	-/-/-
25.	-/6.82/34.09	15.91/22.7/43.1	22.73/45.5/20.5	31.82/25/2.27	29.55/-/-
26.	34.9/56.8/72.73	36.6/34.9/25	15.9/6.82/2.27	9.09/2.27/-	4.55/-/-
27.	-/2.27/31.82	-/25/45.45	15.91/40.9/22.7	34.09/20.5/-	50/11.6/-
28.	-/29.5/61.36	15.9/52.7/38.64	29.55/18.8/-	38.64/-/-	15.91/-/-
29.	11.6/22.7/50	27.7/36.3/45.45	13.6/31.8/4.55	31.8/6.82/-	15.9/2.27/-
30.	16.6/34.1/50	22.7/34.1/38.64	25/20.5/9.09	15.9/11.6/2.27	22.7/-/-
DASH activities: 24. Arm, shoulder or hand pain. 25. Arm, shoulder or hand pain when you performed any specific activity. 26. Tingling (pins and needles) in your arm, shoulder or hand. 27. Weakness in your arm, shoulder or hand. 28. Stiffness in your arm, shoulder or hand. 29. During the past week, how much difficulty have you had sleeping because of the pain in your arm, shoulder or hand? 30. I feel less capable, less confident or less useful because of my arm, shoulder or hand problem.					

Abbreviations: 24–28 questions: 1- none, 2—mild, 3—moderate, 4—severe, 5—extreme; 29 question: 1—no difficulty, 2—mild difficulty, 3—moderate difficulty, 4—severe difficulty, 5—so much I can’t sleep; 30 question: 1—strongly disagree, 2—disagree, 3—neither agree nor disagree, 4—agree, 5—strongly agree.

**Table 7:** A correlation coefficient r with significance p of pain (Visual Analogue Scale, VAS) and Well-being self-evaluation (*n* = 32).

Parameter Correlation (r, *p*)	Pre-Outcomes (I)	Post-Outcomes (II)	Follow-Up Outcomes (III)
Pain score (VAS) vs. Self-esteem	r = 0.37, *p* = 0.03 *	r = −0.20, *p* = 0.26	r = −0.15, *p* = 0.43
VAS vs. Psychological wellness	0.23 (0.02)	0.16 (0.39)	−0.40 (0.02) *
VAS vs. Life satisfaction score	−0.06 (0.74)	0.15 (0.42)	−0.22 (0.23)
VAS vs. Positive Affect	0.06 (0.74)	−0.58 (0.00) *	−0.56 (0.00) *
VAS vs. Negative Affect	0.43 (0.01) *	0.16 (0.39)	0.15 (0.40)
VAS vs. Daily functions	0.09 (0.82)	0.13 (0.48)	−0.04 (0.62)

* Significant differences with *p* < 0.05
